# Supplementary material for: Genetic disease risks can be misestimated across global populations
Source: Genome Biol. 2018 Nov 14;19:179. doi: 10.1186/s13059-018-1561-7 (PMC6234640; doi:10.1186/s13059-018-1561-7)
Supplement: Supplementary file 1 — Table S1. Effects of different p value thresholds for GWAS simulations. (DOCX 49 kb) [file 13059_2018_1561_MOESM1_ESM.docx]

**Table S1**. Effects of different p-value thresholds for GWAS simulations.

| P-value threshold | Allele frequency difference  between Africa and Europe | |
| --- | --- | --- |
|  | Ancestral  risk allele | Derived  risk allele |
| 1x10^-5^ | +10.7% | -8.0% |
| 5x10^-8^ | +12.2% | -8.8% |

GWAS simulation parameters: technology = Affymetrix Genome-Wide Human SNP Array 6.0, sample size = 3500 cases and 3500 controls, study population = EUR, mode of inheritance = additive, prevalence = 0.1, genotype relative risk = 1.211.
