# Supplementary material for: Genetic disease risks can be misestimated across global populations
Source: Genome Biol. 2018 Nov 14;19:179. doi: 10.1186/s13059-018-1561-7 (PMC6234640; doi:10.1186/s13059-018-1561-7)
Supplement: Supplementary file 2 — Table S2. GWAS simulations of dominant, additive, and recessive disease alleles. (DOCX 49 kb) [file 13059_2018_1561_MOESM2_ESM.docx]

**Table S2**. GWAS simulations of dominant, additive, and recessive disease alleles.

| Mode of inheritance | Allele frequency difference  between Africa and Europe | |
| --- | --- | --- |
|  | Ancestral  risk allele | Derived  risk allele |
| Dominant | +19.7% | -2.2% |
| Additive | +10.7% | -8.0% |
| Recessive | +2.9% | -17.9% |

GWAS simulation parameters: technology = Affymetrix Genome-Wide Human SNP Array 6.0, sample size = 3500 cases and 3500 controls, study population = EUR, p-value threshold = 1x10^-5^, prevalence = 0.1, genotype relative risk = 1.211.
