# Supplementary material for: Genetic disease risks can be misestimated across global populations
Source: Genome Biol. 2018 Nov 14;19:179. doi: 10.1186/s13059-018-1561-7 (PMC6234640; doi:10.1186/s13059-018-1561-7)
Supplement: Supplementary file 3 — Figure S1. GWAS simulations that allow effect sizes to vary by population. Figure S2. GWAS simulations with larger effect sizes in Europe. Figure S3. GWAS simulations with larger effect sizes in Africa. Figure S4. Joint site frequency spectra for multiple genotyping technologies. (DOCX 2049 kb) [file 13059_2018_1561_MOESM3_ESM.docx]

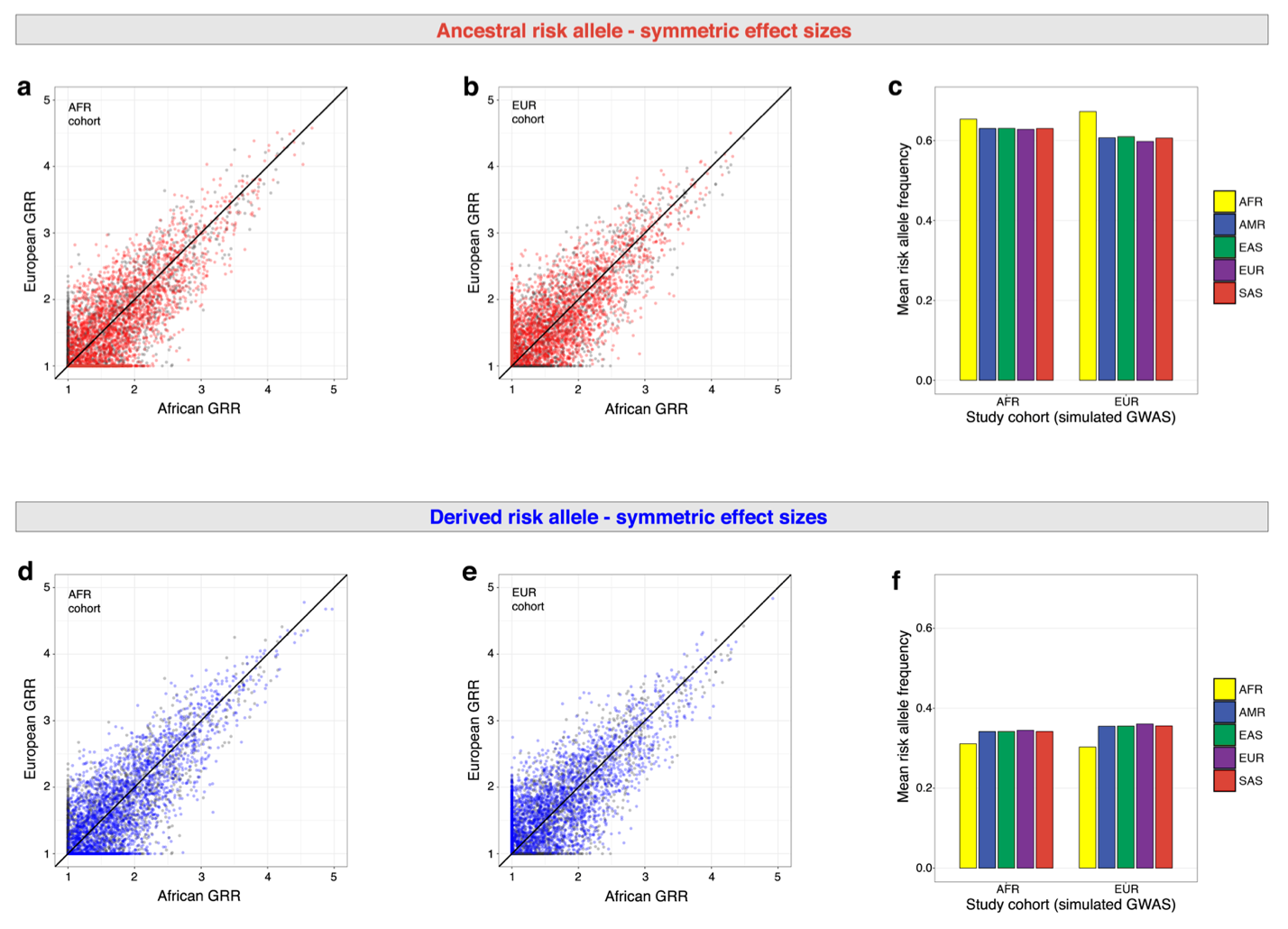


**Figure S1.** GWAS simulations that allow effect sizes to vary by population. Simulation parameters: technology = Affymetrix 6.0, sample size = 3500 cases and 3500 controls, mode of inheritance = additive genetic effects, p-value cutoff = 10^-5^, prevalence = 10%. Panels (**a**), (**b**), and (**c**) show the results of GWAS simulations where the ancestral allele increases risk. Panels (**d**), (**e**), and (**f**) show the results of GWAS simulations where the derived allele increases risk. Panels (**a**), (**b**), (**d**) and (**e**) show representative effect sizes in Europe and Africa, where GRR refers to genotype relative risk. Pre-GWAS effect sizes are indicated by gray points. Post-GWAS effect sizes are indicated by red points (ancestral risk alleles) and by blue points (derived risk alleles). Prior to GWAS simulations, effect sizes are symmetric. Mean risk allele frequencies in different continental populations are shown for each study cohort in panels (**c**) and (**f**)**.**

**
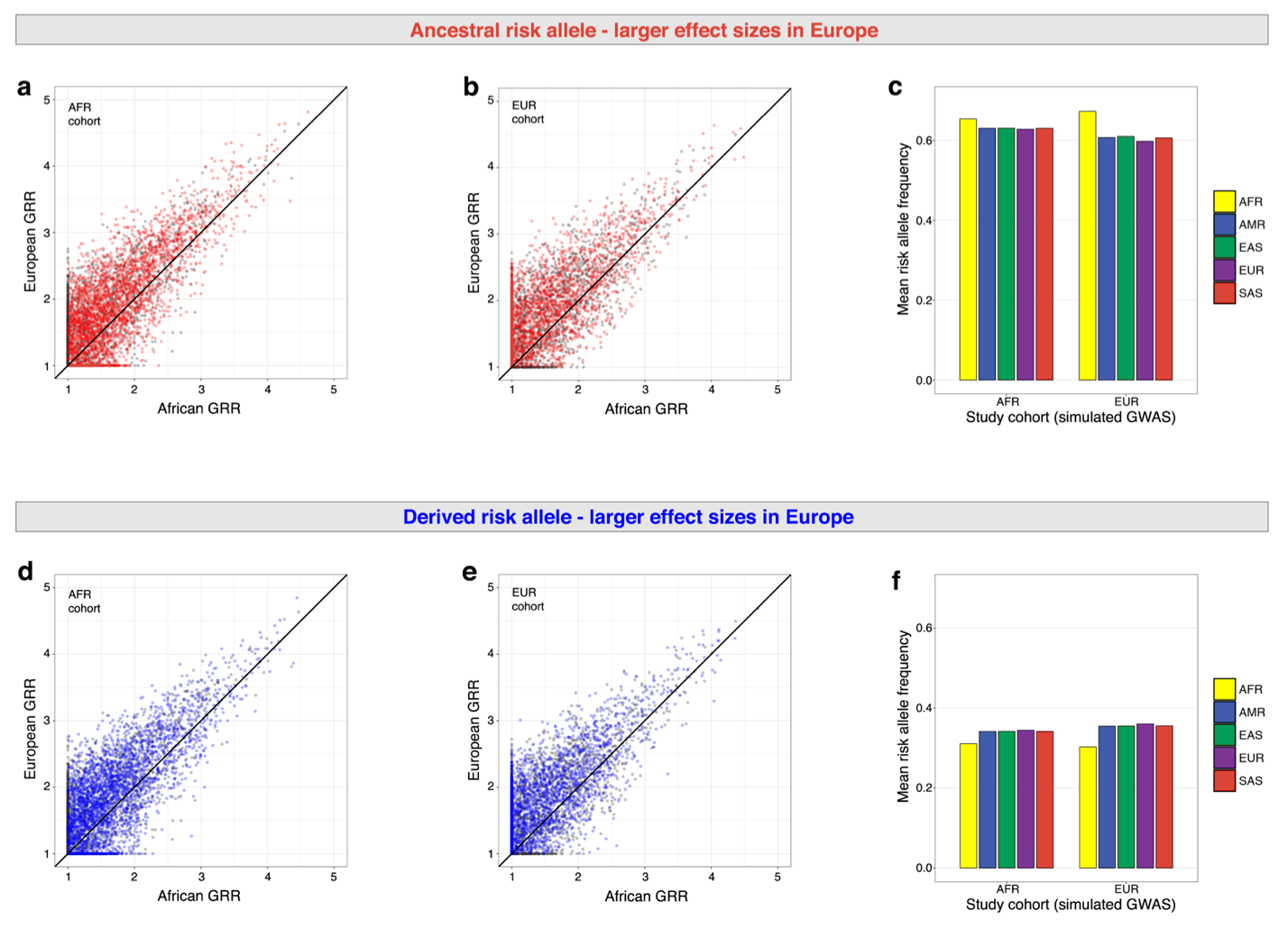
**

**Figure S2.** GWAS simulations with larger effect sizes in Europe. Simulation parameters: technology = Affymetrix 6.0, sample size = 3500 cases and 3500 controls, mode of inheritance = additive genetic effects, p-value cutoff = 10^-5^, prevalence = 10%. Panels (**a**), (**b**), and (**c**) show the results of GWAS simulations where the ancestral allele increases risk. Panels (**d**), (**e**), and (**f**) show the results of GWAS simulations where the derived allele increases risk. Panels (**a**), (**b**), (**d**) and (**e**) show representative effect sizes in Europe and Africa, where GRR refers to genotype relative risk. Pre-GWAS effect sizes are indicated by gray points. Post-GWAS effect sizes are indicated by red points (ancestral risk alleles) and by blue points (derived risk alleles). Prior to GWAS simulations, effect sizes are shifted upwards (i.e. higher in Europe). Mean risk allele frequencies in different continental populations are shown for each study cohort in panels (**c**) and (**f**)**.**

**
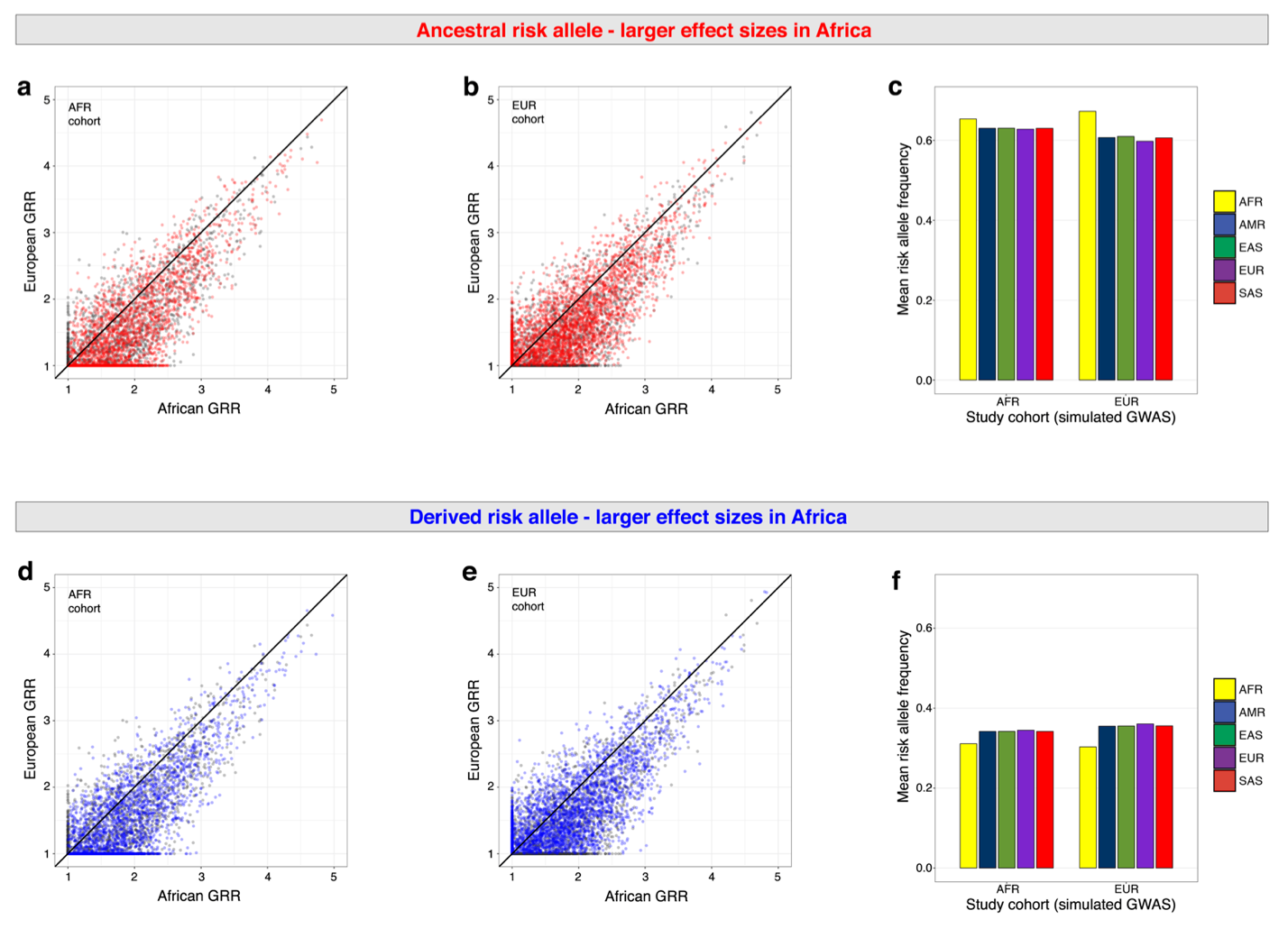
**

**Figure S3.** GWAS simulations with larger effect sizes in Africa. Simulation parameters: technology = Affymetrix 6.0, sample size = 3500 cases and 3500 controls, mode of inheritance = additive genetic effects, p-value cutoff = 10^-5^, prevalence = 10%. Panels (**a**), (**b**), and (**c**) show the results of GWAS simulations where the ancestral allele increases risk. Panels (**d**), (**e**), and (**f**) show the results of GWAS simulations where the derived allele increases risk. Panels (**a**), (**b**), (**d**) and (**e**) show representative effect sizes in Europe and Africa, where GRR refers to genotype relative risk. Pre-GWAS effect sizes are indicated by gray points. Post-GWAS effect sizes are indicated by red points (ancestral risk alleles) and by blue points (derived risk alleles). Prior to GWAS simulations, effect sizes are shifted to the right (i.e. larger in Africa). Mean risk allele frequencies in different continental populations are shown for each study cohort in panels (**c**) and (**f**)**.**


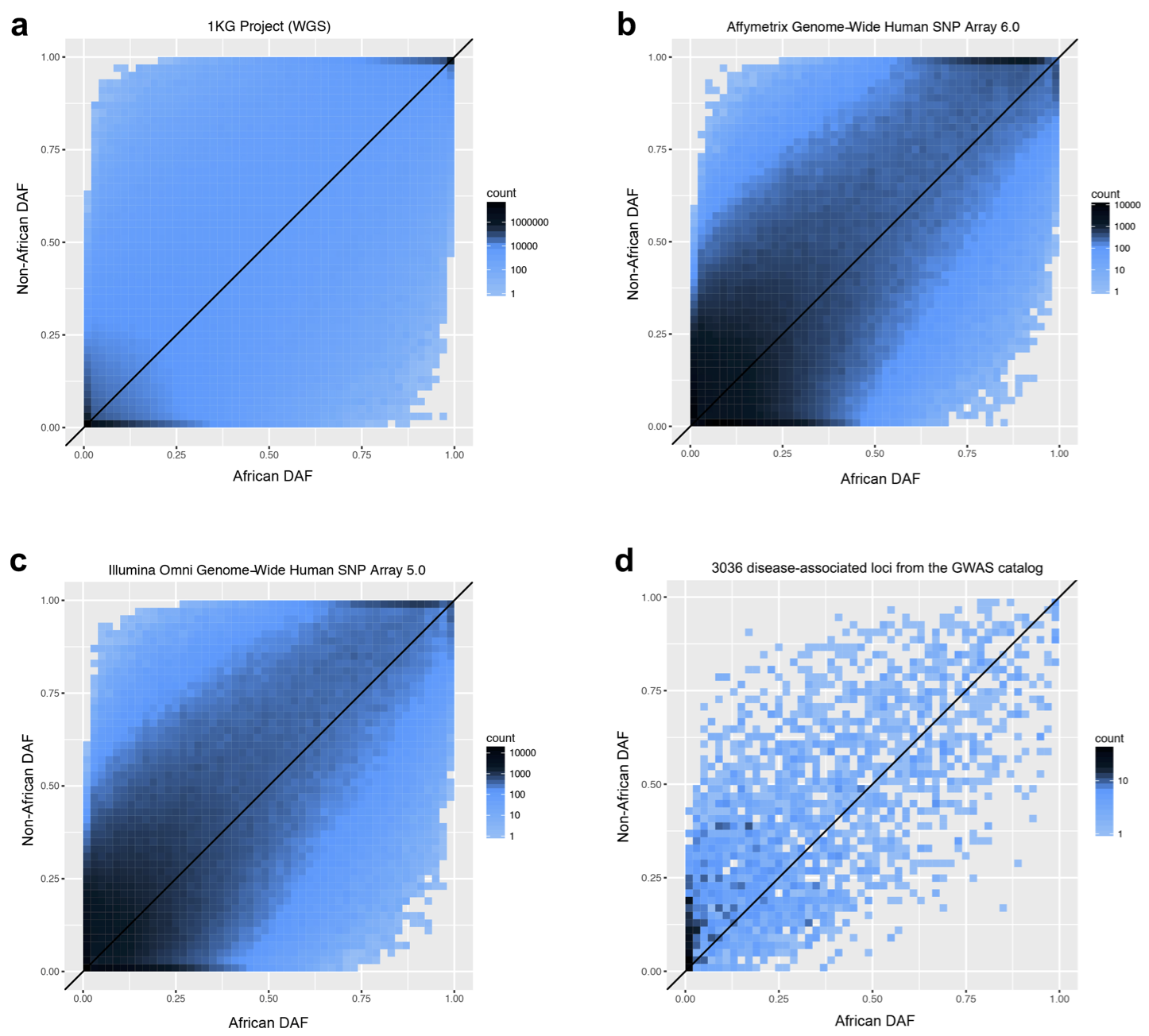


**Figure S4.** Empirical Joint site frequency spectra of for multiple genotyping technologies. DAF are shown in each panel. (**a**) Joint SFS of whole genome sequence (WGS) data. Non-African and African data from the 1000 Genomes Project are shown. Shading indicates counts of SNPs. (**b**) Joint SFS of ascertained SNPs on the Affymetrix Genome-Wide Human SNP Array 6.0. (**c**) Joint SFS of ascertained SNPs on the Illumina Omni 5M microarray. (**d**) Joint SFS of published disease-associated loci from the NHGRI-EBI GWAS Catalog.
